# CT Scans of Patients with 2019 Novel Coronavirus (COVID-19) Pneumonia

**DOI:** 10.7150/thno.45016

**Published:** 2020-03-15

**Authors:** Wei Zhao, Zheng Zhong, Xingzhi Xie, Qizhi Yu, Jun Liu

**Affiliations:** 1 Department of Radiology, The Second Xiangya Hospital, Central South University, No.139 Middle Remin Road, Changsha, Hunan, 410011, P.R. China.; 2 Department of Radiology Quality Control Center, Changsha, Hunan Province, 410011, China.; 3 Department of Radiology, First Hospital of Changsha, Hunan Province, 410005, China.; 4 Changsha Public Health Treatment Center, Hunan Province, 410153, China.

**Keywords:** COVID-19 pneumonia, CT scan, follow up, treatment response

## Abstract

**Rationale:** The increasing speed of confirmed 2019 novel coronavirus (COVID-19) cases is striking in China. The purpose of this study is to summarize the outcomes of patients with novel COVID-19 pneumonia (NCP) at our institution.

**Methods:** In this single-center study, we retrospectively included 118 cases of NCP, from January 16, 2020 to February 4, 2020. The clinical outcomes were monitored up to February 11, 2020. The outcomes of NCP patients were phase summarized at our institution. Three kinds of responses to clinical treatment were defined and evaluated: 1) good, symptoms continually improved; 2) fair, symptoms not improved or relapsed; 3) poor, symptoms aggravated. The risk factors, including basal clinical characteristics, CT imaging features, and follow-up CT changes (no change, progress, and improvement) related to poor/fair outcomes, were also investigated.

**Results:** Six patients were improved to no-emergency type, 2 remained the same, and 2 progressed to fatal type. Besides, 13 patients progressed from the common type group to the emergency group (3 in fatal type and 10 in severe type). Forty-two (35.6%) patients were discharged with a median hospital stay of 9.5 days (range, 4.0-15.0 days). Thus, the numbers in different responses were, 73 patients in good response group (4 emergency cases, 69 no-emergency cases), 28 in fair response group (3 emergency cases, 25 no-emergency cases), and 17 in poor response group (3 emergency cases, 14 no-emergency cases). No patient has died in our hospital to date. The median duration of progress observed from CT scans was 6 days (range, 2-14 days). The progression in abnormal imaging findings indicate a poor/fair response, whereas the alleviated symptoms seen from CT suggest a good response.

**Conclusion:** Most cases are no-emergency type and have a favorable response to clinical treatment. Follow-up CT changes during the treatment can help evaluate the treatment response of patients with NCP.

## Introduction

2019 novel coronavirus (COVID-19) pneumonia (NCP), first reported in Wuhan (Hubei province, China), has drawn intense attention around the world 1. Import cases have been reported in Thailand, Japan, South Korea, and US 2-5, and the number of involved countries is increasing. Given the potential of the global outbreak, the World Health Organization (WHO) has declared the COVID-19 outbreak as a Public Health Emergency of International Concern (PHEIC) 6. The number of laboratory-confirmed COVID-19 cases has surpassed that of severe acute respiratory syndrome coronavirus (SARS-CoV) 7, and there is no clear trend to decline. As of February 16, 2020, 70548 laboratory-confirmed cases were reported in 31 provinces (municipalities and regions) in China, including 10644 fatal cases, 1770 death cases, and 7264 suspected cases 8. The mortality of 2019-nCoV is lower than that of SARS-CoV and Middle East respiratory syndrome coronavirus (MERS-CoV) 7. However, the panic caused by this disease is not less than the previous two diseases.

Specific onset symptoms (*i.e.*, fever), clinical features (*i.e.*, leukopenia), and epidemic characteristics (*i.e.*, exposure history to Wuhan or close contact history with confirmed cases) can substantially help in diagnosing the disease 9, 10. However, due to the lack of a better understanding of the inherent biological characteristics of COVID-19, no treatment scenarios or medicine can effectively control the disease. Symptomatic and routine antiviral treatments seem to be the only choice for clinicians. This circumstance leads to a long-term admission stay and prevents many suspected cases from being diagnosed and treated in the hospital. Moreover, few confirmed cases may progress rapidly after admission and die of multiple organ failure 11. Therefore, a better understanding of the risk factors related to poor outcomes could provide a better prospect for improving the clinical scenarios.

The outcome features of patients (*i.e.*, median discharge time, mortality) with NCP have been briefly reported recently 11, 12. However, the detailed features of NCP patients with a good or poor clinical response have not been well studied. Also, the relationship between basal clinical characteristics and the response or outcomes remains unclear. Computed tomography (CT) is being increasingly emphasized in the diagnosis and evaluation of response in clinical practice 13-15, and has the potential to provide valuable information in reflecting the extent of the disease. The follow-up CT changes during the clinical treatment and whether there is a correlation between the treatment outcomes and radiologic features of NCP patients have not been well documented yet.

In this context, the purpose of this study was to summarize the outcomes of NCP patients in our institution. We investigated the risk factors, including basal clinical characteristics and radiographic features related to poor outcomes.

## Materials and Method

This study was approved by the Medical Ethical Committee (Approval Number.KL-2020001), which waived the requirement for patients' informed consent referring to the CIOMS guidelines.

### Patients

From January 16, 2020 to February 4, 2020, a search of the electronic system and picture achieving and communication system (PACS) was performed by one of our authors in our institution. The inclusion criteria were as follows: 1) patients with laboratory-confirmed 2019-nCoV; 2) patients underwent CT scan more than one time. Diagnosis of 2019-nCoV was determined according to the following three methods: isolation of 2019-nCoV or at least two positive results by real-time reverse-transcription-polymerase chain reaction (RT-PCR) assay for 2019-nCoV or a genetic sequence that matches 2019-nCoV. Finally, a total of 118 consecutive laboratory-confirmed 2019-nCoV patients (60 females, 58 males; mean age, 44.06 years ± 13.62 [SD]; median age, 42.5 years; age range, 2-75 years), who underwent serial CT scans, were included. All available clinical and epidemic characteristics were collected. We characterized patients into four types: mild, common, severe and fatal based on the guidelines of 2019-nCoV (Trial Version 5) 16, proposed by the China National Health Commission. As the treatment regimens between different types were different, we divided the included patients into two groups: no-emergency group (mild type and common type) and emergency group (severe type or fatal type). The interval of follow-up CTs ranged from 2-7 days.

### Imaging Technique

All included patients were scanned using one of the following three scanners: GE, HiSpeed-Dual, 64-slice LightSpeed VCT (GE Medical Systems) and Somatom emotion (Siemens Medical Solutions). The acquisition parameters were as follows: 120 kVp; 100-200 mAs; pitch, 0.75-1.5; and collimation, 0.625-5 mm. All imaging data were reconstructed by using a medium sharp reconstruction algorithm with a thickness of 0.625-5mm. CT images were acquired in the supine position at full inspiration for all patients.

### Image Interpretation

All chest CT scans were reviewed blindly and independently by two radiologists (with 5 and 15 years of experience) in consensus. All images were viewed on both lung (width, 1500 HU; level, -700 HU) and mediastinal (width, 350 HU; level, 40 HU) settings. Fourteen image features including the presence of ground-glass opacities (GGO), consolidation, mixed GGO and consolidation, centrilobular nodules, architectural distortion, cavitation, tree-in-bud, bronchial wall thickening, reticulation, subpleural bands, traction bronchiectasis, intrathoracic lymph node enlargement, vascular enlargement in the lesion, and pleural effusions were evaluated. The detailed definitions of the above features were well documented in the previous study 17. We evaluated four types of distributions: the craniocaudal distribution (upper lung predominant, lower lung predominant, or no craniocaudal predilection), the transverse distribution (central, peripheral, or no transverse predilection); the lung region distribution (unilateral or bilateral) and the scattering distribution (focal, multifocal or diffuse). Focal was defined as a single lesion of abnormality, multifocal as more than one lesions, and diffuse as the involvement of most of the volume of one lung lobe. A CT score system was used to evaluate the extent of disease 18. We defined three imaging changes: no change, progress change, and improvement change. No change referred to no obvious changes presented in chest CT. Progress change referred to the presence of new lesions or the presence of extent involvement area during the treatment. Improvement change referred to the continually absorbed abnormities. We also evaluated the duration of imaging progress, which was calculated from the time of baseline CT or the time of CT showing new lesions to that of the CT showing abnormal imaging findings.

### Treatment

All suspected and confirmed cases were transferred to 2019-nCoV designated hospitals. We followed the therapeutic principles based on the guidelines of 2019-nCoV (Trial Version 5) proposed by the China National Health Commission 16. The basic treatment included the symptomatic treatment, recombinant human interferon α2b (aerosol inhalation) and antiviral treatment, such as lopinavir or ritonavir tablets (500 mg twice daily, orally). Corticosteroid treatment and antibiotic treatment were used where appropriate. Invasive mechanical ventilation treatment was used for fatal cases. Extracorporeal membrane oxygenation (ECMO) was used for life support.

### Definition of Clinical outcomes

Discharge and release quarantine criteria: 1) body temperature returned to normal for more than 3 days; 2) respiratory symptoms significantly relieved; 3) abnormal imaging findings substantially resolved; 4) viral clearance, *e.g.* negative nucleic acid test for two consecutive respiratory pathogens (sampling interval ≥1 day). We defined 3 kinds of responses to clinical treatment: 1) good, symptoms continually improved; 2) fair, symptoms not improved or relapsed; 3) poor, symptoms aggravated.

### Statistical Analysis

Continuous variables were presented as mean (SD) and compared with the Mann-Whitney U test; categorical variables were described as frequency rates and percentages (%) and compared by χ² test or Fisher's exact test between normal and emergency groups. A two-sided P of less than 0.05 was considered statistically significant. All statistical analyses were performed using the SPSS software (version 24.0).

## Results

### Clinical characteristics

At the time of admission,108 patients (8 in mild type, 100 in common type; male 53, female 55) were placed in the no-emergency group, whereas the other 10 patients (all in severe type, male 5, female 5) were categorized as the emergency group. As shown in **Table 1**, 69.5% of the patients were less than 50 years old in our cohort and 2 were young cases, 2 and 11 years of age. Not surprisingly, both patients were related to a family outbreak. With respect to the epidemic history, 92 (78.0%) patients had an exposure history to Wuhan and 24 (20.3%) patients were exposed to confirmed patients or patients without a diagnosis but had previous exposure. Eight (6.8%) patients denied any specific epidemic history and 15 (12.7%) patients were related to a family outbreak (more than 2 patients were confirmed in one family). In terms of onset symptoms, 91 (77.1%) patients had an onset symptom of fever and 24 (20.3%) had a symptom of fever before admission. The other two common symptoms were cough (54.2%) and myalgia or fatigue (19.5%). Other rare onset symptoms are listed in **Table 1.** It is of note that 3 (2.5%) patients had no onset symptoms before admission but had a close contact history with confirmed cases.

### CT findings and follow-up changes

Ground-glass opacity (GGO) (101, 85.6%) or mixed GGO and consolidation (74, 62.7%) were detected in most patients with NCP. CT images also showed vascular enlargement in the lesions in most patients (92, 78.0%). Intrathoracic lymph node enlargement and pleural effusions were rare imaging features in patients with NCP. The abnormal CT findings were more likely to be peripheral distribution (97, 82.2%), bilateral involvement (94, 79.7%), and multifocal (65, 55.1%). The frequency of other evaluated imaging features is presented in **Table 2**. Cavitation, tree-in-bud were absent in our cohort; 8 patients were identified as negative findings on CT images. The mean score of the lung involvement at baseline was 6.18. Sixty-four (54.2%) patients presented improvement based on CT changes, whereas CT changes progressed in 49 (41.5%) patients. Six patients remained the same on CT findings during the treatment and 5 of 6 patients had no abnormal CT findings at baseline. Among the 49 patients with progress in CT changes, the median duration of imaging progress was 6 days (range, 2-14 days). One patient presented new lesions in the latest CT scan and the progress time was not calculated. In patients with an improvement from follow-up CT, GGO lesions were reduced directly or changed to consolidation and then to fibrosis.

### Clinical outcomes

As of February 11, 2020, out of all 8 severe cases, 6 were improved to no-emergency type, 2 remained the same and 2 progressed to fatal type. Besides, 13 patients progressed from the common type group to the emergency group (3 in fatal type and 10 in severe type). Forty-two (35.6%) patients were discharged with a median hospital stay of 9.5 days (range, 4.0-15.0 days). Collectively, the numbers in different response groups were: 73 patients in good response group (4 emergency cases, 69 no-emergency cases), 28 in fair response group (3 emergency cases, 25 no-emergency cases), and 17 in poor response group (3 emergency cases, 14 no-emergency cases). None has died in our hospital to date.

### Basal clinical characteristics, radiographic features, and treatment response

**Figure 1:**
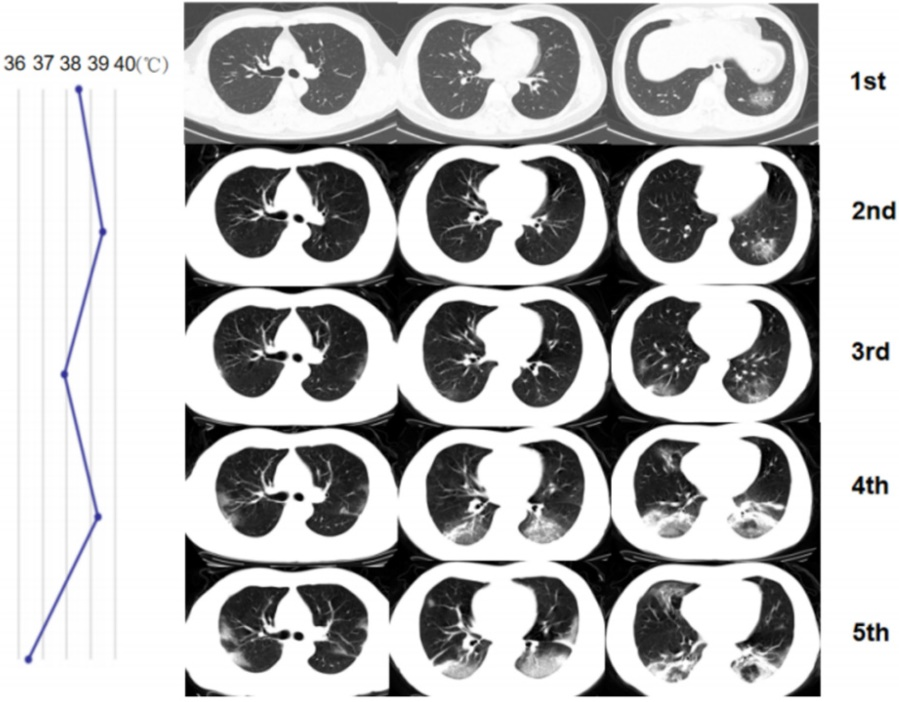
A 35-year-old man with confirmed 2019-nCoV infection, common type at baseline. Patient had a short-term exposure history to Wuhan and a close contact history with confirmed cases. The onset symptom was fever (38 °C). During the treatment, the follow-up CT changes presented a progress type (the involved area of lesions enlarged). The patient became severe type to date. The graph on the left presented the temperature changes during the treatment. The dot represented the highest temperature on the day of corresponding CT scan time. The temperature fluctuation indicates poor response (CT scans were performed on January 24, 26 and 30, 2020 and February 1 and 7, 2020 respectively).

**Figure 2:**
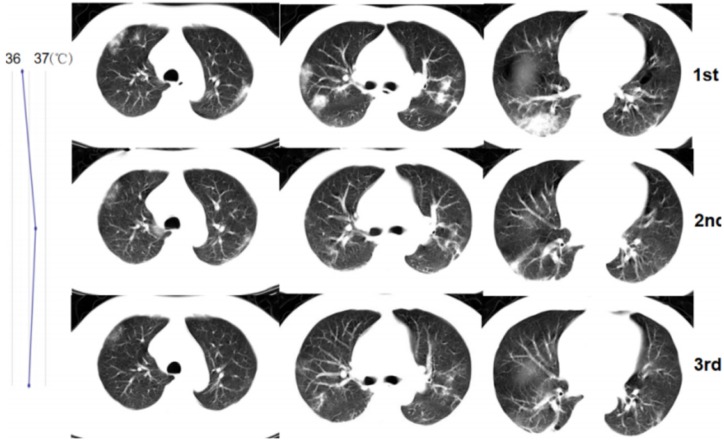
A 53-year-old man with confirmed 2019-nCoV infection, common type at baseline. Patient had an exposure history to Wuhan and the onset symptom of fever and cough. During the treatment, the follow-up CT changes presented an improvement type (the imaging lesions continually absorbed). The residual lesions were fibrosis. The patient was discharged with a hospital stay of 9 days. The graph on the left presented the temperature changes during the treatment. The dot represented the highest temperature on the day of corresponding CT scan time (CT scans were performed on January 26, 29, 2020 and February 1, 2020, respectively).

**Figure 3:**
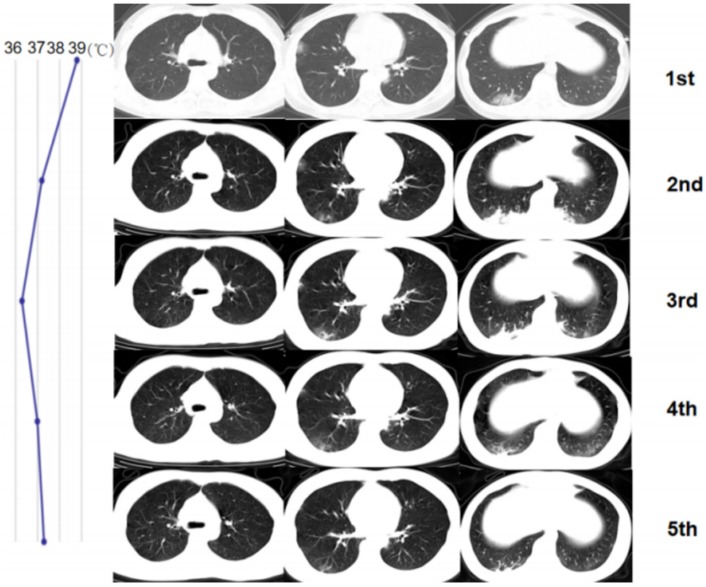
A 32-year-old woman with confirmed 2019-nCoV infection, common type at baseline. Patient had an exposure history to Wuhan and the onset symptom of fever and cough. During the treatment, the follow-up CT changes presented a progress type (the enlargement of lesions and then narrowed later). The imaging progress duration was 3 days. The residual lesions were fibrosis. The patient was discharged with a hospital stay of 11 days. The graph on the left presented the temperature changes during the treatment. The dot represented the highest temperature on the day of corresponding CT scan time (CT scans were performed on January 27 and 30, 2020 and February 2, 5 and 8, 2020 respectively). Note that CT finding changes did not match the temperature changes (good response clinically).

We divided the patients into good response and poor/fair response groups. The differences in basal clinical characteristics and radiographic features between the two groups were analyzed and compared. No significant differences were found between the two groups in clinical characteristics such as sex, age, exposure history, onset symptoms, underlying disease, and clinical type at baseline (**Table 1**). The types, features, and distribution of lesions showed no significant differences between the two groups (**Table 2**). Based on the follow-up CT changes, no change, progress change, and improvement change showed statistically significant differences between the two groups (**Table 2**). The progress trend in abnormal imaging findings might indicate a poor/fair response (**Figure 1**), whereas the alleviated symptoms seen in the follow-up CT scans might indicate a good response **(Figure 2)**. However, the imaging changes may not necessarily always match the changes in clinical symptoms. Five patients in the good response group but 1 patient in the poor/fair group presented an improvement in follow-up CT **(Figure 3)**. Thus, the follow-up CT scans could help us evaluate the treatment response in most cases. For the 48 patients with progressive CT changes, we further compared the progress duration time in the two groups. The results showed that the duration of imaging progress was longer in the poor/fair group than that in the good group (*P =* 0.009).

## Discussion

In this study, we summarized the clinical characteristics, radiographic features, and clinical outcomes of 118 patients with NCP. The potential risk factors related to poor response were also investigated. We believe that the follow-up CT changes during the treatment could help us effectively evaluate the treatment response of patients with NCP.

COVID-19 has attracted the attention of most people all around the world. The confirmed cases are still increasing with incredible speed in China. Clinicians equipped with epidemic prevention supplies from all around China continue to come to Wuhan to control the disease, indicating a serious situation. COVID-19 is biologically close to SARS-CoV with an R0 of approximately 2.2 9. The mortality of COVID-19 is 2.5%, based on the statistics report on Feb 16, 2020 in China, but the cure rate is reported to be only 15.4% in China. No fully proven antiviral scenario for the coronavirus exists to date. Early detection, diagnosis, and early isolation and treatment remain the basic effective strategies to fight the outbreak of COVID-19 19. Early detection and diagnosis can substantially control the transmission and reduce the confirmed cases with unknown routes of transmission (8 cases in our cohort) and family outbreak cases (15 cases in our cohort). Although the onset symptoms vary, fever (77.1%) and cough (53.4%) are the most common ones, which is consistent with previous studies 12, 14 and might help screen the suspected cases. However, the incubation period might be as long as 24 days 20. In this regard, it is of note that 2 patients had no onset symptoms in our cohort; however, the epidemic history and abnormal CT findings indicated the 2019-nCoV infection. This highlights the importance of scrutinizing the epidemic exposure history of patients in the clinic.

Patients with older age or underlying disease are prone to have a poor prognosis 12. However, the death of a 34-year old doctor with NCP must be considered earnestly. In our cohort, no one has died to date and the cure rate is 35.6%, which may have contributed to the relatively low rate of emergency type (8.5%). We analyzed and compared the differences in basal clinical characteristics and radiographic features between the good response and poor/fair response groups. No specific factors were identified as risk factors for poor response, including age, underlying disease, and baseline clinical type. In this context, it is difficult for clinicians to take measures or change treatment scenarios to prevent potential progression by evaluating baseline conditions for a patient. In our study, 13 patients subsequently progressed from a non-emergency type to an emergency type (3 in fatal type and 10 in severe type). Therefore, further studies of the lung microenvironment and the response of the immune system against 2019-nCoV infection are urgently needed.

CT scanning plays a vital role in the early detection and diagnosis of COVID-19 and can be considered as a clinical diagnostic modality 16. Several typical imaging features (*i.e.* GGO, mixed GGO and consolidation, bilateral distribution) can identify the high suspected patients 13, 21. However, the baseline CT findings do not help in identifying the patients who might progress later. It is clear that the follow-up CT changes can effectively evaluate and predict the later response. Of note is the observation that the imaging changes might not always match the changes in clinical symptoms. In our study, 1 patient presented an improvement in imaging change but poor response. The patient with type 2 diabetes was one of 13 patients who progressed from the common type group into the emergency group. The assumption was that the underlying disease condition might affect the response. On the contrary, 5 patients presented a progress imaging change but a good response. This may be explained by the improvement in clinical symptoms that preceded imaging. Another interesting finding was that the duration of imaging progress might be valuable in predicting the prognosis of patients with NCP. Our results emphasize the importance of follow-up CT scans.

Nevertheless, the study has several limitations. First, this was a single-center study and only 118 patients (most were imported cases) with confirmed COVID-19 were included. A multicenter study and/or including more cases, especially more emergency types, might provide more information on the clinical outcomes of COVID-19. Second, some important laboratory test results, like the viral load (cycle threshold value), were not analyzed in our study due to the limited data availability. Comprehensive acquisition and analysis of these data in our ongoing studies are expected to provide us with more information and help design better treatment strategies.

In conclusion, many cases in our cohort were no-emergency type and had a favorable response to clinical treatment. Most importantly, the follow-up CT changes during the treatment could help evaluate the treatment response of patients with NCP.

## Figures and Tables

**Table 1:** Basal epidemic and clinical features of the patients

Basal Characteristics	All Patients (n=118)	Good Response (n=73)	Poor/Fair Response (n=45)	*P*
Sex				0.738
Male	60 (50.8)	38 (52.1)	22 (48.9)	
Female	58 (49.2)	35 (47.9)	23 (51.1)	
Age				
Mean age ± SD	44.06±13.62	43.08±12.50	45.64±15.28	0.283
Range	2-75			
≤20	3 (2.5)			
21-40	48 (40.7)			
41-50	31 (26.3)			
51-60	18 (15.3)			
61-70	17 (14.4)			
≥70	1 (0.8)			
Epidemic History				
Direct exposure history	92 (78.0)	59 (80.8)	33 (73.3)	0.340
Indirect exposure history	24 (20.3)	13 (17.8)	11 (24.4)	0.384
Family outbreak	15 (12.7)	7 (9.6)	8 (17.8)	0.195
No exposure history	8 (6.8)	4 (5.5)	4 (8.9)	0.474
Onset symptoms				
Fever	91 (77.1)	55 (75.3)	36 (80.0)	0.559
Cough	64 (54.2)	42 (57.5)	22 (34.4)	0.360
Myalgia or fatigue	23 (19.5)	12 (52.2)	11 (24.4)	0.286
Sore throat	13 (11.0)	8 (11.0)	5 (11.1)	0.980
Diarrhoea	6 (5.1)	3 (4.1)	3 (6.7)	0.539
Nausea and vomiting	3 (2.5)	1 (1.4)	2 (4.4)	0.303
Headache	4 (3.4)	2 (2.7)	2 (4.4)	0.619
Dyspnoea	1 (0.8)	0 (0)	1 (2.2)	0.201
More than one symptom	80 (67.8)	45 (61.6)	33 (73.3)	0.193
None	3 (2.5)	2 (2.7)	1 (2.2)	0.862
Underlying disease				
Cardiovascular and cerebrovascular disease	18 (15.3)	13 (17.8)	5 (11.1)	0.326
Endocrine system and rheumatology disease	9 (7.6)	5 (6.8)	4 (8.9)	0.685
Surgery History	9 (7.6)	5 (6.8)	4 (8.9)	0.685
Digestive system disease	4 (3.4)	1 (1.4)	3 (6.7)	0.123
Respiratory system disease	4 (3.4)	1 (1.4)	3 (6.7)	0.123
Urinary system disease	2 (1.7)	0 (0)	2 (4.4)	0.069
None	81 (68.6)	52 (71.2)	29 (64.4)	0.440
Clinical type at baseline				0.329
Mild type	8 (6.8)	5 (6.8)	3 (6.7)	
Common type	100 (84.7)	64 (87.7)	36 (80.0)	
Severe type	10 (8.5)	4 (5.5)	6 (13.3)	
Clinical outcome				**0.000**
Remained in hospital	76 (64.4)	34 (46.6)	42 (93.3)	
Discharged	42 (35.6)	39 (53.4)	3 (6.7)	

Note—Except for age (mean with SD in parentheses) data are number with percentage in parentheses

**Table 2:** CT features and follow-up CT changes in patients

	All Patients (n=118)	Good Response (n=73)	Poor/Fair Response (n=45)	*P*
Image findings				
GGO	101 (85.6)	65 (89.0)	36 (80.0)	0.174
Consolidation	64 (54.2)	39 (53.4)	25 (55.6)	0.821
Mixed GGO and consolidation	74 (62.7)	47 (64.4)	27 (60.0)	0.632
Centrilobular nodules	24 (20.3)	17 (23.3)	7 (15.6)	0.311
Architectural distortion	23 (19.5)	12 (16.4)	11 (24.4)	0.286
Bronchial wall thickening	21 (17.8)	14 (19.2)	7 (15.6)	0.617
Reticulation	48 (40.7)	25 (34.2)	23 (51.1)	0.070
Subpleural bands	42 (35.6)	26 (35.6)	16 (35.6)	0.995
Traction bronchiectasis	41 (34.7)	21 (28.8)	20 (44.4)	0.082
Intrathoracic lymph node enlargement	1 (0.8)	1 (1.4)	0 (0)	0.430
Vascular enlargement	92 (78.0)	56 (76.7)	36 (80.0)	0.676
Pleural effusions	14 (11.9)	11 (15.1)	3 (6.7)	0.170
Craniocaudal distribution				0.533
Upper lung predominant	6 (5.1)	2 (2.7)	4 (8.9)	
Lower lung predominant	50 (42.4)	32 (43.8)	18 (40.0)	
No craniocaudal distribution	54 (45.8)	34 (46.6)	20 (44.4)	
Transverse distribution				0.158
Central	3 (2.5)	0 (0)	3 (6.7)	
Peripheral	97 (82.2)	61 (83.6)	36 (80.0)	
No transverse distribution	10 (8.5)	7 (9.6)	3 (6.7)	
Lung region distribution				0.884
Unilateral	16 (13.6)	9 (12.3)	7 (15.6)	
Bilateral	94 (79.7)	59 (80.8)	45 (77.8)	
Scattering distribution				0.791
Focal	12 (10.2)	6 (8.2)	6 (13.3)	
Multifocal	65 (55.1)	40 (54.8)	25 (55.6)	
Diffuse	33 (28.0)	22 (30.1)	11 (24.4)	
Extent of the lesion (Mean ± SD)	6.18 ± 4.47	6.15 ± 4.36	6.22 ± 4.68	0.984
Imaging features changes				**0.000**
No change^*^	6 (5.1)	6 (8.2)	0 (0)	
Progress change^*^	49 (41.5)	5 (6.8)	44 (97.8)	
Improvement change^*^	63 (53.4)	62 (84.9)	1 (2.2)	
No abnormal findings	8 (6.8)	5 (4.2)	3 (2.5)	0.969

Note—Except for extent for the lesion (mean with SD in parentheses) data are number with percentage in parentheses. * means the statistically different between any two groups with a corrective P value (Bonferroni method).
